# Comprehensive Insights Into the Multi-faceted Manifestations of COVID-19: A Narrative Review

**DOI:** 10.7759/cureus.63493

**Published:** 2024-06-30

**Authors:** Sairama Gollapudi, Vilas Chimurkar

**Affiliations:** 1 Department of Medicine, Jawaharlal Nehru Medical College, Datta Meghe Institute of Higher Education and Research, Wardha, IND; 2 Department of Anatomy, Jawaharlal Nehru Medical College, Datta Meghe Institute of Higher Education and Research, Wardha, IND

**Keywords:** multi-system impact, covid-19 clinical presentations, psychological effects, extrapulmonary manifestations, sars-cov-2, covid-19, long covid, corona virus symptoms, extra-pulmonary manifestation, covid 19

## Abstract

The emergence of severe acute respiratory syndrome coronavirus-2 (SARS-CoV-2) and the ensuing COVID-19 pandemic had far-reaching and multifaceted effects on global health. This paper provides a comprehensive overview of the physical, extrapulmonary, and psychological manifestations associated with COVID-19. It highlights the wide-ranging impact of the virus on various organ systems, including the respiratory, cardiovascular, renal, gastrointestinal, ocular, dermatologic, and nervous systems. Additionally, it explores the complex connections between COVID-19 infection and neuropsychiatric symptoms, shedding light on the potential underlying mechanisms. The paper also delves into the phenomenon of "long COVID," a condition characterized by persistent symptoms extending well beyond the disease's acute phase. It discusses the diverse and often debilitating symptoms that individuals with long COVID may experience, encompassing physical, cognitive, and psychological aspects. The complexity and variability of long COVID underscore the challenges it poses to healthcare professionals and the importance of ongoing research to understand its underlying mechanisms. Furthermore, the paper touches on the current state of knowledge regarding the aetiology of long COVID and the various approaches to symptom management and treatment. While a definitive cure remains elusive, efforts are underway to alleviate the burden of long COVID through pharmacological interventions, physical therapy, cognitive-behavioral therapy, and support networks. This paper comprehensively explores COVID-19's far-reaching effects, emphasizing the need for a holistic and interdisciplinary approach to understanding and managing the diverse manifestations of this global health challenge. Ongoing research and collaborative efforts are essential in addressing the complex and evolving nature of COVID-19 and its aftermath.

## Introduction and background

A new member of the coronavirus family, Coronaviridae, is the severe acute respiratory syndrome coronavirus-2 (SARS-CoV-2). The coronavirus disease 19 or COVID-19, a highly contagious and sometimes fatal illness, is linked to this virus, drawing interest worldwide. Since it first appeared, SARS-CoV-2 has spread widely, posing an incomparable public health threat [1]. The World Health Organization (WHO) declared the COVID-19 outbreak a pandemic on March 11, 2020, due to the virus' rapid propagation. Since the H1N1 influenza pandemic in 2009, this announcement was the first time the WHO had categorized an epidemic as a pandemic [2]. The Coronavirus has impacted many individuals. When RT-PCR (reverse transcription PCR of nasopharyngeal and oropharyngeal swab samples) was positive, it manifested as a range of symptoms in the patients [3]. Symptoms may have changed with different COVID-19 variants and could vary depending on vaccination status. Some of the affected populations were also asymptomatic. The symptoms varied for different patients during Covid, post-covid and post-vaccination [4]. They are the physical and psychological signs and symptoms. Patients tested positive for the second time even after being vaccinated. The course of the illness typically involves a gradual improvement in symptoms over a few days to weeks after the onset of the first signs. Within this timeframe, most people tend to make a full recovery from the acute infection, regaining their health and returning to their normal daily activities. However, a subset of individuals experiences a more prolonged and lingering set of symptoms, even after COVID-19's acute phase has passed [5]. This condition has been termed "long COVID". It encompasses a range of persisting symptoms that continue for weeks or even months beyond the initial infection [6]. After infection, an individual with post-COVID problems may experience various symptoms for weeks, months, or even years. Sometimes, the symptoms may even disappear or resurface [7].

## Review

Search methodology

We undertook a systematic search through PubMed and CENTRAL in December 2021 using keywords such as "coronavirus" and "extrapulmonary" (((Coronavirus [Title/Abstract]) OR (COVID [Title/Abstract])) OR (long covid [Title/Abstract])) AND ("extrapulmonary" [MeSH Terms]) AND (psychological [Title/Abstract])) OR ("covid-19" [MeSH Terms]). We additionally searched for key references from bibliographies of the relevant studies. The search was updated in February 2023.

**Figure 1:**
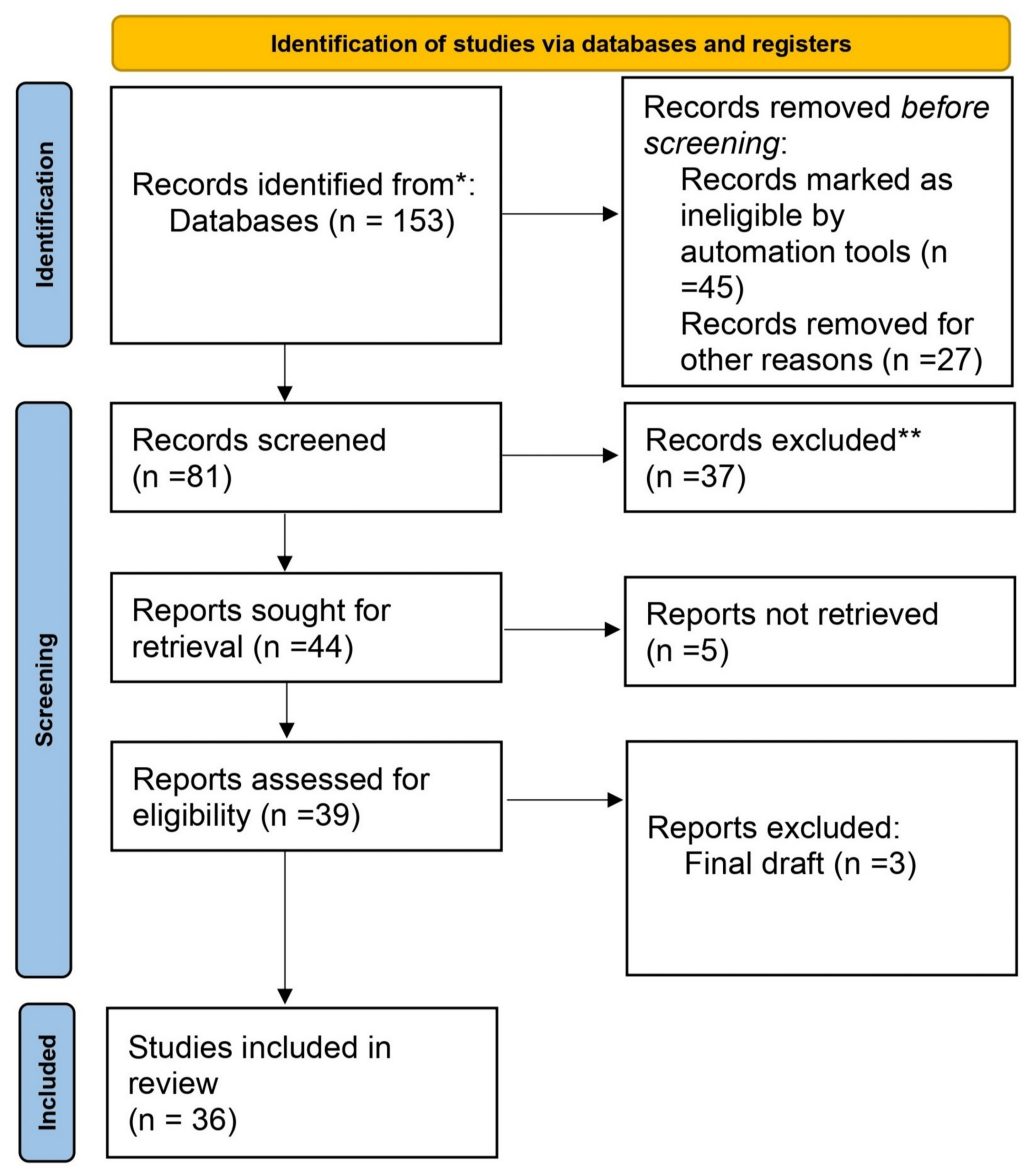
Prisma flow diagram for literature search Adapted from- PRISMA_2020_flow_diagram_new_SRs_v1

Based on the title and abstract, one reviewer independently evaluated the retrieved papers against the inclusion criteria. Approximately 30% of these papers were also assessed by another reviewer to confirm their inclusion. Any disagreements were resolved through discussion. We extracted data on the physical, extrapulmonary, and psychological effects of coronavirus disease from the research. The PRISMA flow diagram for the literature search, which provides a visual summary of the screening process, is presented in Figure 1 below [8].

Physical symptoms

Since 2002, there have been three significant outbreaks of Coronavirus, a class of viruses known to cause respiratory illness. SARS-CoV, which infected over 8,000 individuals and killed close to 800 people globally, was the source of the initial outbreak. MERS-CoV, which infected over 2,500 individuals and killed over 850 people globally, was the source of the second outbreak. The most recent outbreak, which started in December 2019, was brought on by the SARS-CoV-2, a new coronavirus. SARS-CoV-2 has caused approximately 36.5 million infections and over 1 million fatalities globally as of February 2023. Coronaviruses are thought to originate in bats. However, they can mutate and adapt to infect other animals, such as camels (MERS-CoV) and civets (SARS-CoV) [9]. Rarely, these viruses may transfer to people, resulting in an epidemic. The SARS-CoV-2 virus is believed to have started in bats, moved on to another animal, perhaps a pangolin, and finally jumped to us. The virus was believed to be passed from person to person by respiratory aerosols released when an infected person coughed or sneezed. The SARS-CoV-2 infection has no particular treatment. However, some things may be done to stop the infection from spreading.

There is a similarity in the symptoms of the COVID-19 infection and those of acute respiratory distress syndrome, which comprise fever or chills, cough, shortness of breath or issues with respiration, fatigue, muscle or body aches, headache, new loss of taste or smell, sore throat, congestion or runny nose, nausea or vomiting, and diarrhoea [10].

Extrapulmonary symptoms

As is well known, COVID-19's manner of manifestation has parallels to both the Middle East Respiratory Syndrome (MERS) of 2012 and the severe acute respiratory syndrome (SARS) of 2003, with a significant focus on the pulmonary system [11]. However, it is essential to recognize that this viral disease's effects reach beyond the respiratory system's confines and extend their influence to various other vital organs within the human body. While the initial presentation of COVID-19 may be respiratory, its far-reaching impact can lead to intricate involvement in multiple organ systems, leading to potentially severe consequences. This expanded impact raises concerns and highlights the need for comprehensive medical attention and a deeper understanding of the complexities involved in managing and treating this novel coronavirus infection. With such a vast array of potential organ involvement, researchers and healthcare professionals are continuously exploring the nuances of COVID-19's pathophysiology to provide the most effective care and mitigate the broader implications it may have on human health. Through ongoing research and clinical observations, we can gain a more comprehensive understanding of the virus's diverse manifestations, enabling us to implement targeted interventions and optimize patient outcomes across various organ systems.

Consequently, the battle against COVID-19 transcends mere respiratory concerns, and a holistic approach to managing this global health challenge becomes crucial to safeguarding the well-being of individuals affected by the virus [12]. COVID-19 has the potential to exert its influence on multiple organ systems that extend far beyond its initial focus on the lungs or pulmonary system. This novel coronavirus infection can have widespread implications, affecting crucial organs such as the heart, belonging to the cardiovascular system, the kidneys within the renal system, the liver, which falls under the hepatic system, the gastrointestinal tract, the eyes, the skin, and even the intricate nervous system. This wide-ranging impact underscores the complexity of the disease and the need for a comprehensive understanding of its pathophysiology. As COVID-19 infiltrates various organ systems, its diverse manifestations demand the attention and expertise of medical professionals across specialities to provide optimal care and effectively manage the complexities presented by this global health challenge. By acknowledging the potential involvement of multiple organs, researchers can further explore the mechanisms through which COVID-19 affects these vital systems, thereby facilitating the development of targeted treatment strategies and interventions to minimize the adverse effects and maximize positive patient outcomes. As the medical community continues to delve into the intricate interactions between COVID-19 and different organ systems, valuable insights can be gained, equipping us with the knowledge needed to combat this virus effectively and safeguard public health on a global scale [13].

Cardiac manifestation

SARS-CoV-2, the virus due to which COVID-19 is caused, has been found to instigate both direct and indirect complications affecting the cardiovascular system, leading to a spectrum of potential cardiac issues [14]. Among these complications are myocardial injury, acute coronary syndromes, cardiomyopathy, arrhythmias, and cardiogenic shock, all of which can significantly impact an individual's cardiovascular health and overall prognosis [15].​​​​​​

Myocardial Injury and Acute Coronary Syndromes

Myocardial injury is a prominent cardiovascular complication in COVID-19 patients, characterized by elevated cardiac troponin levels. This condition may arise from direct viral invasion of myocardial cells or from systemic inflammation and cytokine storms. Acute coronary syndromes, including myocardial infarction, can occur due to increased thrombotic activity and endothelial dysfunction induced by the virus.

Cardiomyopathy and Heart Failure

Cardiomyopathy, particularly stress-induced (Takotsubo) cardiomyopathy, has been observed in COVID-19 patients. The inflammatory response triggered by the infection can weaken the heart muscle, leading to heart failure. This condition requires careful management to prevent further deterioration of cardiac function.

Arrhythmias

Arrhythmias, including atrial fibrillation, ventricular tachycardia, and bradyarrhythmias, are common in COVID-19 patients. These can result from direct viral effects on cardiac tissue, electrolyte imbalances, or the impact of systemic inflammation. Monitoring and managing arrhythmias are crucial to prevent severe cardiac events.

Cardiogenic Shock

Cardiogenic shock, a severe condition where the heart cannot pump enough blood to meet the body's needs, can develop in COVID-19 patients. It often results from extensive myocardial injury or acute heart failure, necessitating urgent medical intervention to support cardiac function and ensure adequate perfusion.

Cardiovascular Risk Factors

Extensive research, including comprehensive meta-analyses, has revealed that COVID-19 patients are more susceptible to certain cardiovascular risk factors. The prevalence of hypertension, cardio-cerebrovascular disease, and diabetes mellitus among COVID-19 patients is notably high, at 17.1%, 16.4%, and 9.7%, respectively. These conditions are significantly more common in severe cases requiring I.C.U. admission, indicating their role in influencing disease severity and patient outcomes.

Acute Cardiac Injury

A meta-analysis identified that approximately 8% of COVID-19 patients experience acute cardiac injury. The incidence of acute cardiac injury is about 13 times higher in I.C.U./severe cases compared to non-ICU cases.

This underscores the importance of vigilant cardiac monitoring in managing COVID-19 patients, particularly those with severe disease. This acute injury to the heart further complicates the clinical course of the disease and underscores the importance of closely monitoring cardiac health in individuals battling COVID-19 [15].

Renal manifestation

In patients suffering from COVID-19, a sudden and significant complication often observed is acute kidney injury (AKI) [16]. This rapid deterioration in kidney function is linked to an increase in patient mortality and morbidity. The causes of AKI in COVID-19 patients are diverse and include direct viral invasion, systemic inflammation, and hemodynamic instability.

While the frequency of AKI among COVID-19 patients can vary, it is a common complication. The SARS-CoV-2 virus has the ability to directly invade renal cells by attaching to the angiotensin-converting enzyme 2 (ACE2) receptors, which are plentiful in the kidneys. This direct viral invasion can result in damage to the tubules and inflammation in the interstitial tissue.

The systemic inflammatory response associated with COVID-19 contributes to damage to the endothelium, increased vascular permeability, and subsequent impairment of renal function. Hemodynamic instability, including hypotension and hypoxia, often seen in severe COVID-19 cases, can further intensify kidney injury. Hypoperfusion and hypoxemia can lead to acute tubular necrosis, a common form of AKI. In a notable study conducted by Wang et al., it was revealed that approximately 4% of individuals diagnosed with COVID-19 experienced acute kidney injury. Furthermore, the administration of nephrotoxic medications during the treatment of COVID-19 can also contribute to renal dysfunction.

This highlights the importance of careful monitoring of renal function in COVID-19 patients and underscores the complex interplay of factors contributing to AKI in these patients. It also emphasizes the need for ongoing research to better understand the pathophysiology of AKI in the context of COVID-19 and to develop effective strategies for its prevention and management [17].

Gastrointestinal manifestation

Gastrointestinal (GI) symptoms have emerged as a noteworthy component of the clinical presentation of the disease [18]. These symptoms, which include nausea, diarrhoea, abdominal pain, and vomiting, can often manifest prior to the onset of respiratory symptoms. This early manifestation may serve as an initial indicator of infection, potentially aiding in the prompt identification and management of COVID-19 cases. Although these symptoms may not be directly linked to an increase in mortality, they have been associated with a prolonged duration of illness in certain cases [19].

The occurrence of GI symptoms among COVID-19 patients is not uncommon. A study involving a cohort of 138 hospitalized patients revealed that a subset of these patients experienced GI symptoms: 10% reported episodes of nausea and diarrhoea, 5% experienced abdominal pain, and 3% had episodes of vomiting. These findings underscore the importance of acknowledging GI symptoms as a potential manifestation of COVID-19. Interestingly, in 10% of these patients, nausea and diarrhoea were reported one to two days prior to the onset of respiratory symptoms. This sequence of symptom onset suggests that GI symptoms could potentially serve as an early warning sign of infection [17].

The underlying mechanisms contributing to the GI involvement in COVID-19 are multifaceted. The SARS-CoV-2 virus has the ability to infect the gastrointestinal tract by binding to the angiotensin-converting enzyme 2 (ACE2) receptors, which are highly expressed in the enterocytes of the small intestine. This interaction facilitates the entry and replication of the virus within the GI tract, leading to the manifestation of symptoms such as diarrhoea and nausea. In addition, the systemic inflammatory response triggered by the infection can contribute to abdominal pain and vomiting. The presence of these symptoms can complicate the clinical course of COVID-19, particularly in patients with pre-existing gastrointestinal conditions. Therefore, the recognition and management of these symptoms are integral to the comprehensive care of COVID-19 patients.

Hepatic manifestation

Hepatotoxicity, or liver damage, has become a significant concern in patients suffering from severe COVID-19 [20]. This concern stems from the observation that the SARS-CoV-2 virus can affect liver function, leading to noticeable disruptions in liver-related metabolic processes [21]. Investigations into liver function among COVID-19 patients have revealed critical insights into the extent of liver involvement. In a detailed study involving 99 individuals with confirmed COVID-19, about 43% exhibited abnormal liver enzyme levels, indicating liver impairment. Among these patients, 98% displayed decreased levels of albumin, a key protein essential for various bodily functions such as maintaining oncotic pressure and serving as a carrier for numerous substances. This decrease suggests a disruption in hepatic synthesis, a critical function of the liver. Further examination of liver enzymes in the study participants revealed elevated levels of aspartate aminotransferase (AST) in 35%, alanine aminotransferase (ALT) in 28%, and bilirubin in 18% of the cases. Elevated AST and ALT are indicative of hepatocellular injury, while increased bilirubin levels point to cholestasis or impaired bile flow, both of which are signs of liver damage. These abnormalities reflect the significant impact that COVID-19 can have on liver health, particularly in severe cases [22].

The liver's involvement in COVID-19 may be due to several factors, including direct viral effects on hepatocytes and bile duct cells, systemic inflammation, and the effects of hypoxia and sepsis. The liver damage observed in these patients highlights the importance of monitoring liver function in COVID-19, especially since liver impairment can complicate the clinical management and prognosis of affected individuals. Comprehensive management strategies are needed to address liver health in COVID-19 patients, incorporating careful monitoring of liver enzymes and albumin levels, as well as supportive care to mitigate the effects of liver damage. This understanding underscores the broader systemic impact of COVID-19 and the need for an integrated approach to manage the complex manifestations of the disease.

Ocular manifestation

While respiratory symptoms are the primary focus of COVID-19, ocular manifestations have also been reported in a subset of cases [23]. These manifestations can include conjunctivitis, which is commonly referred to as pink eye, as well as more severe conditions such as retinitis, anterior uveitis, and optic neuritis. Conjunctivitis involves inflammation of the conjunctiva, leading to redness, irritation, and discharge from the eyes. It is considered one of the milder ocular symptoms associated with COVID-19 but can serve as an early sign of the infection.

Retinitis, an inflammation of the retina, can lead to vision problems and, if left untreated, potential vision loss. Anterior uveitis is the inflammation of the uvea, the middle layer of the eye, which can cause pain, light sensitivity, and blurred vision. Optic neuritis, the inflammation of the optic nerve, can result in visual impairment and may signify a more severe impact of the virus on the central nervous system.

Despite these reported ocular symptoms, there is still a significant gap in comprehensive data regarding their prevalence and clinical course. This lack of extensive data is likely due to under-recognition and under-reporting, as eye symptoms may be overshadowed by more severe systemic manifestations of COVID-19. Moreover, the focus on respiratory complications in the early stages of the pandemic may have contributed to less attention being paid to ocular issues.

However, recent studies and case reports have started to shed light on the presence of ocular symptoms in COVID-19 patients. For instance, a study published in JAMA Ophthalmology found that among hospitalized COVID-19 patients, a notable percentage reported eye-related symptoms, with conjunctivitis being the most common. This suggests that while ocular symptoms may not be as prevalent as respiratory symptoms, they are nonetheless an important aspect of the disease that warrants attention [24].

Dermatologic manifestation

Dermatologic symptoms have been observed in a subset of COVID-19 patients, highlighting the virus's potential to affect the skin. A single-centre observational study in Italy revealed that 20% of hospitalized COVID-19 patients who had no recent drug exposure that could account for the symptoms exhibited various dermatologic manifestations. These skin symptoms include erythematous rash, vesicular lesions, and urticaria (hives). An erythematous rash is characterized by redness of the skin caused by increased blood flow to the capillaries. Vesicular lesions are small, fluid-filled blisters that can appear on the skin's surface, while urticaria presents as itchy, raised welts that can vary in size and shape [25].

The study’s findings suggest that dermatologic symptoms may be a relatively common yet underrecognized aspect of COVID-19. These skin manifestations can appear at different stages of the infection, sometimes even before the onset of respiratory symptoms, and can persist for varying durations. The exact mechanism by which SARS-CoV-2 causes these skin changes is still under investigation, but it is believed to involve immune-mediated responses, direct viral effects on the skin, and the body’s inflammatory reaction to the infection.

Despite these observations, there is still a significant need for further research to better understand the prevalence, characteristics, and clinical implications of dermatologic symptoms in COVID-19 patients. The variety of skin manifestations and their potential overlap with reactions to medications or other infections complicate the clinical picture. Although there is little information on the subject, erythematous rash, vesicular lesions, and urticaria are currently documented manifestations [26].

Documented cases of COVID-19-related dermatologic symptoms have included patients with chilblain-like lesions (also known as "COVID toes"), livedo reticularis (a lace-like purplish discolouration of the skin), and other inflammatory skin conditions. These symptoms can provide clues to the diagnosis, particularly in patients who might not exhibit prominent respiratory symptoms initially.

Neurological manifestation

Recently, a growing body of evidence highlights the wide-ranging neurological complications observed in patients with COVID-19. Research suggests that the SARS-CoV-2 virus can impact the central nervous system (CNS) and the peripheral nervous system (PNS), contributing to a spectrum of neurological manifestations [27]. Among the most commonly reported CNS manifestations of COVID-19 are headache, acute cerebrovascular disease (commonly known as stroke), dizziness, and encephalopathy. A retrospective study involving 214 confirmed COVID-19 patients revealed that a significant proportion, approximately 36.4% of the subjects, collectively experienced one or more of these neurological symptoms, underscoring the complexity and diversity of neurological involvement in COVID-19 cases. Additionally, an analysis of 214 patients with severe COVID-19 demonstrated that approximately 36% experienced various neurologic symptoms. These neurological complications can range from mild to severe, and in some cases, they can pose serious threats to patients' health and even lead to fatal outcomes.

Despite the mounting evidence of neurological complications, the precise mechanisms through which SARS-CoV-2 triggers these neurological effects are not yet fully clarified. However, researchers speculate that the virus may directly infect cells within the nervous system or indirectly induce damage through inflammatory responses or the formation of blood clots. The intricate interplay between the virus and the nervous system underscores the need for further research to comprehend these mechanisms comprehensively [27]. Anorexia, anosmia, and ageusia are common minor neurological symptoms in hospitalized COVID-19 patients, including headache, dizziness, myalgia, or tiredness.

Psychiatric and psychological manifestations

The COVID-19 pandemic has significantly affected mental health, leading to a wide range of psychiatric and psychological symptoms [28]. The neuropsychiatric manifestations associated with COVID-19 are not yet fully understood, but several theories offer potential explanations. One hypothesis suggests that the inflammation resulting from the virus or the body's immune response can cause damage to brain cells, leading to neuropsychiatric symptoms. This inflammation may be part of the broader systemic inflammatory response seen in severe COVID-19 cases. Another theory posits that the propensity of COVID-19 to induce blood clots can limit blood flow to the brain, contributing to neurological issues such as cognitive impairment and acute delirium. Additionally, there is growing evidence indicating that SARS-CoV-2 might directly infect neural cells through mechanisms involving ACE2 receptors, leading to direct brain inflammation and injury [29].

The intense fear of infection, combined with the psychological strain of quarantine and social distancing, has exacerbated various mental health conditions. Patients have reported experiencing confusion, delirium, depression, fatigue, insomnia, post-traumatic stress disorder (PTSD), anxiety, and obsessive-compulsive symptoms [30]. A study involving 461 patients revealed that 16.3% exhibited clinically significant anxiety, 26.5% showed signs of depression, 33.4% experienced insomnia, and 11.7% had suicidal ideation [31]. These statistics underscore the considerable mental health burden posed by COVID-19, highlighting the need for vigilant mental health monitoring and supportive care in both acute and post-acute phases of the disease.

Beyond the clinical neuropsychiatric symptoms, the broader psychological impact of the COVID-19 pandemic has been profound. The pervasive uncertainty and fear surrounding the virus, alongside the disruptive effects of quarantine and social distancing, have led to elevated levels of stress and anxiety in the general population. Many individuals have reported significant changes in their sleep patterns, appetite, and general mental well-being, characterized by increased fear and worry. The economic fallout from the pandemic has further exacerbated psychological distress, with widespread job losses and financial instability significantly contributing to heightened levels of anxiety and depression. The resulting financial pressure has strained households and affected individuals' mental health, leading to increased feelings of insecurity and helplessness. Furthermore, there has been a notable rise in substance use, as some people have turned to alcohol and drugs as coping mechanisms to deal with the heightened stress and anxiety caused by the pandemic. This increase in substance use has the potential to create a vicious cycle of addiction, further complicating mental health issues and impeding recovery efforts. Addressing these broader psychological impacts requires a holistic approach that integrates mental health support with public health strategies, ensuring that individuals receive the necessary psychological and social support to navigate the ongoing challenges posed by the pandemic.

Long COVID

Long COVID, a term used to describe the lingering effects that persist for extended periods, spanning weeks to months, after an individual has been infected with the SARS-CoV-2 virus, has garnered significant attention in the medical community [6]. According to the U.S. Centers for Disease Control and Prevention (CDC), this collection of longer-term symptoms can be remarkably diverse and encompass various physical, psychological, and neurological manifestations, varying in intensity and duration among affected individuals [8].

The respiratory symptoms, such as persistent shortness of breath and cough, can be particularly debilitating. These symptoms may be a result of residual lung damage caused by the virus or a manifestation of a post-viral syndrome. In some cases, individuals may develop a condition known as dysautonomia, where the autonomic nervous system does not function properly, leading to issues with heart rate, blood pressure, and breathing.

Fatigue or post-exertional malaise is another common symptom that can significantly impact an individual’s daily functioning. This is not just ordinary tiredness but a profound lack of energy that can make even simple tasks seem insurmountable. It’s important to note that this fatigue is not alleviated by rest and can be exacerbated by physical or mental exertion.

Cognitive difficulties, often referred to as “brain fog,” can also be a significant issue. This can manifest as problems with memory, attention, and cognitive speed. It can affect an individual’s ability to work, drive, and carry out other daily activities [32]. The exact cause of these cognitive issues is not yet fully understood, but they may be related to the impact of the virus on the brain or a result of the general inflammation caused by the illness.

The persistent symptoms of long COVID, such as chest or stomach pain, joint or muscle pain, and a pins-and-needles sensation, can also be distressing. These may be due to the inflammatory response of the body to the virus. In some cases, these symptoms may be a manifestation of an autoimmune response triggered by the virus.

Gastrointestinal disturbances, such as diarrhoea, can also occur. This may be due to the virus’s impact on the gut microbiome. Changes in the gut microbiome have been linked to a range of health issues, including immune response, metabolism, and mental health.

Sleep disturbances and fluctuations in body temperature, such as fever, are also commonly reported. These may be linked to the body’s ongoing immune response to the virus.

The complexity of long COVID is further highlighted by the fact that the combination of these symptoms can vary widely among individuals [33]. Moreover, the severity of these symptoms can significantly differ among individuals, ranging from mild discomfort to profound debilitation [34]. Some may experience only one or two mild symptoms, while others may grapple with multiple severe symptoms simultaneously. This variability makes it challenging to predict the course of the illness and to develop effective treatment strategies.

In addition to the physical consequences, long COVID can also encompass long-term psychological and neurological symptoms, further adding to the complexity of this condition. These manifestations can impact an individual's mental health, contributing to mood changes and potentially exacerbating existing psychological conditions. Furthermore, neurological symptoms may arise, affecting various aspects of the central and peripheral nervous systems. Such symptoms may include dizziness upon standing, often referred to as lightheadedness, and perceptible changes in an individual's sensory experiences, such as alterations in smell or taste. In some instances, long COVID has been linked to changes in menstrual period cycles in affected individuals [35].

The multifaceted nature of long COVID poses numerous challenges for medical professionals and patients. Comprehensive research efforts are continually underway to unravel this condition's underlying mechanisms and develop effective strategies for diagnosis, management, and treatment. Gaining a more profound understanding of long COVID and its impact on diverse bodily systems enables healthcare providers to tailor treatment plans to address the unique needs and complexities faced by those living with the aftermath of SARS-CoV-2 infection. Moreover, providing appropriate support and care for the psychological and neurological aspects of long COVID is essential in ensuring the overall well-being of those affected by this perplexing and evolving condition [36].

The exact aetiology behind the persistence of long COVID remains elusive at present, with researchers continuing their quest to unravel the complex mechanisms involved in this prolonged condition. The prevailing hypothesis suggests that the SARS-CoV-2 virus, which initially caused the acute infection, may be responsible for inflicting damage to various organs and tissues within the body. This damage, in turn, triggers a cascade of inflammatory responses and other physiological changes that contribute to the emergence of long-term symptoms. Notably, the body's immune response also emerges as a significant factor in developing long COVID. While a critical defence against viral invaders, the immune system can sometimes mount a response that perpetuates inflammation and tissue damage even after the initial infection has been controlled. This immune-mediated phenomenon is suspected to play a pivotal role in perpetuating symptoms long after the acute phase of COVID-19 has subsided.

Despite ongoing research, a definitive cure for long COVID has not yet been identified. Nonetheless, medical professionals have focused on developing effective symptom management strategies to improve the quality of life for those affected. These treatment approaches encompass various modalities tailored to address specific long-term symptoms. Pharmacological interventions are often employed to target symptoms such as pain, fatigue, and other distressing manifestations. Additionally, physical therapy is frequently utilized to enhance muscle strength and endurance, helping individuals regain functional capacity and alleviate physical limitations. Recognizing the potential psychological toll of long COVID and cognitive-behavioural therapy has proven beneficial in assisting patients in coping with anxiety and depression that may arise as a consequence of the extended illness. Support groups also offer a valuable resource for those grappling with long COVID, fostering a sense of community and connection among individuals who share similar experiences.

Continual research is a pivotal aspect in comprehending and effectively managing long COVID. Scientists are diligently working to decipher the intricate mechanisms underpinning this condition, pinpoint potential risk factors, and explore innovative treatment modalities. Additionally, efforts are underway to develop preventive measures to thwart the development of long COVID in those afflicted with SARS-CoV-2 infection [37].

## Conclusions

With each new breakthrough in our understanding of COVID and long COVID, the medical community takes significant strides towards developing comprehensive approaches that can better tackle the intricacies of these persistent health conditions. This ongoing research instills optimism and relief for individuals grappling with the uncertainties of COVID and long COVID, as it promises more targeted and effective management strategies. As we continue to gain insights into the mechanisms and manifestations of these conditions, patients can look forward to improved outcomes and an enhanced quality of life, bringing us closer to a future where these challenging conditions can be managed with greater precision and care.
